# Estimating Chlorophyll Content of Leafy Green Vegetables from Adaxial and Abaxial Reflectance

**DOI:** 10.3390/s19194059

**Published:** 2019-09-20

**Authors:** Fan Lu, Zhaojun Bu, Shan Lu

**Affiliations:** 1 Key Laboratory of Geographical Processes and Ecological Security in Changbai Mountains, Ministry of Education, School of Geographical Sciences, Northeast Normal University, Renmin 5268, Changchun 130024, China; luf785@nenu.edu.cn (F.L.); buzhaojun@nenu.edu.cn (Z.B.); 2 Jilin Provincial Key Laboratory for Wetland Ecological Processes and Environmental Change in the Changbai Mountains, Institute for Peat and Mire Research, Northeast Normal University, Renmin 5268, Changchun 130024, China

**Keywords:** adaxial and abaxial, reflectance, chlorophyll, vegetation index, partial least squares (PLS)

## Abstract

As a primary pigment of leafy green vegetables, chlorophyll plays a major role in indicating vegetable growth status. The application of hyperspectral remote sensing reflectance offers a quick and nondestructive method to estimate the chlorophyll content of vegetables. Reflectance of adaxial and abaxial leaf surfaces from three common leafy green vegetables: Pakchoi var. Shanghai Qing (*Brassica chinensis* L. var. Shanghai Qing), Chinese white cabbage (*Brassica campestris* L. ssp. *Chinensis* Makino var. *communis* Tsen et Lee), and Romaine lettuce (*Lactuca sativa* *var longifoliaf. Lam*) were measured to estimate the leaf chlorophyll content. Modeling based on spectral indices and the partial least squares regression (PLS) was tested using the reflectance data from the two surfaces (adaxial and abaxial) of leaves in the datasets of each individual vegetable and the three vegetables combined. The PLS regression model showed the highest accuracy in estimating leaf chlorophyll content of pakchoi var. Shanghai Qing (R^2^ = 0.809, RMSE = 62.44 mg m^−2^), Chinese white cabbage (R^2^ = 0.891, RMSE = 45.18 mg m^−2^) and Romaine lettuce (R^2^ = 0.834, RMSE = 38.58 mg m^−2^) individually as well as of the three vegetables combined (R^2^ = 0.811, RMSE = 55.59 mg m^−2^). The good predictability of the PLS regression model is considered to be due to the contribution of more spectral bands applied in it than that in the spectral indices. In addition, both the uninformative variable elimination PLS (UVE-PLS) technique and the best performed spectral index: MDATT, showed that the red-edge region (680–750 nm) was effective in estimating the chlorophyll content of vegetables with reflectance from two leaf surfaces. The combination of the PLS regression model and the red-edge region are insensitive to the difference between the adaxial and abaxial leaf structure and can be used for estimating the chlorophyll content of leafy green vegetables accurately.

## 1. Introduction

The human consumption of leafy green vegetables has been increasing due to lifestyle changes in recent years, and hence the nutrition and health status of leafy green vegetables on the market is of important to consumers [1,2,3]. Chlorophyll, as the primary pigment of leafy green vegetables, plays a major role in assessing the health status of vegetables. The nutritional status of leafy green vegetables can also be monitored via quantifying chlorophyll content because most of the nitrogen is incorporated in leaf chlorophyll [4,5,6,7]. Hence, there is a need for accurate, efficient, and practical methodologies to estimate leaf chlorophyll content [8,9,10]. Non-destructive remote determination of leaf chlorophyll content by reflectance permits a way to quickly measure chlorophyll variation in leaves and avoid destructive and expensive traditional laboratory-based chlorophyll content measurements [11,12].

Many reflectance-based vegetation indices (VIs) that include a single band or multiple bands have been developed to estimate the chlorophyll content of plants. Most of the indices utilize the reflectance in the feature bands, such as near infrared (NIR) (750–870 nm), green (550 nm), and red (660–670 nm) [3,13,14]. Additionally, more sensitivity of reflectance in the red-edge region than the reflectance in the other bands to chlorophyll content of vegetation has been recognized for decades [13,14,15,16,17], and the red-edge bands have been widely used for biophysical parameters at leaf and canopy levels [18]. The red edge is the region of sharp change in vegetation reflectance spectra. It occurs between wavelengths of 680–750 nm, where the reflectance changes from very low in the chlorophyll red absorption region to very high in the NIR because of leaf and canopy scattering [19].

Spectral indices mostly focus only on a few bands, which makes it difficult to construct a unified index to remotely estimate leaf chlorophyll content across different plant species or different growth stages. In contrast, the partial least squares (PLS) regression is a so-called full-spectrum technique that reduces the large number of measured collinear spectral variables to a few non-correlated latent variables or factors while maximizing co-variability to the variable(s) of interest [20,21,22]. Some researchers have demonstrated that the PLS regression model could make the prediction more robust and accurate in the quantitative analysis of biochemical compositions in plants [23,24,25]. However, the original PLS method uses all available wavebands, which are not always informative. Some researchers used an uninformative variable elimination PLS (UVE-PLS) approach for the selection of the informative bands before PLS modeling [24,25,26]. UVE-PLS is a method for variable selection based on an analysis of regression coefficients of PLS [26], which can remove lots of useless variables and retain the primary valuable-information-containing variables. The UVE-PLS method has been widely applied in analytical chemistry, and satisfactory prediction results were obtained [23]. However, limited research has been conducted to estimate the leaf chlorophyll content with reflectance. 

The adaxial leaf side is traditionally considered in reflectance measurements assuming that the reflectance captured by remote sensors is mostly from the adaxial leaf. Recently, dorsiventral spectral leaf data are gaining attention, such as the development of dorsiventral radiative transfer models [27], the detection of disease and water status in wheat [28], and leaf air pollution estimation [29]. Only very few studies have focused on the leaf chlorophyll content estimation by dorsiventral leaf reflectance [30,31,32]. However, these studies merely utilized a linear regression analysis with only two or three wavebands to build a valid spectral index, but did not involve more wavebands, such as in PLS regression analysis. In addition, most remote reflectance measurement methods have worked on quantifying the chlorophyll content of grasses, crops, deciduous trees, and coniferous trees [12,33,34,35,36,37], whereas fewer studies have been conducted on green leaves vegetables [3].

One of the aims of the present study was to investigate the validation of the spectral indices and PLS regression model in estimating the leaf chlorophyll content of vegetables with dorsiventral leaf reflectance. The prediction performances of leaf chlorophyll content by the two methods were compared, and the approach that was insensitive to the adaxial and abaxial leaf structure was determined. The other aim was to select the most informative spectral bands retained in each of the two methods, spectral indices, and PLS, for estimating chlorophyll content of three different green leaves vegetables.

## 2. Materials and Methods

### 2.1. Plant Materials

Three different species of common green leafy vegetables with different leaf colors: Pakchoi var. Shanghai Qing (*Brassica chinensis* L.var. Shanghai Qing) with dark green leaves, Chinese white cabbage (*Brassica campestris L. ssp. chinensis Makino*) with green leaves and Romaine lettuce (*Lactuca sativa var longifoliaf. Lam*) with middle green leaves were sampled from vegetable greenhouse in suburban farmland and used as our experimental materials. In order to obtain a wide range of variation of chlorophyll content of the leaf samples, the vegetables (pakchoi var. Shanghai Qing (n = 66), Chinese white cabbage (n = 60), and Romaine lettuce (n = 62)) at different growing stages were harvested from vegetable greenhouse. The chlorophyll content of the three species vegetables is shown in Table 1. It can be seen that the vegetable leaf samples covered a very wide range (7.20 to 557.57 mg m^−2^) of chlorophyll variation (corresponding to different leaf color change from yellow to dark green). The minimum chlorophyll content of Romaine lettuce was significantly greater than that of pakchoi var. Shanghai Qing and Chinese white cabbage, mainly because the leaves of Romaine lettuce were very thin and easy to be damaged, and it is hard to get samples with the very low chlorophyll content.

About 70% of the samples were used for the model calibration and the remainder were used for model validation.

### 2.2. Reflectance Measurements

**Figure 1:**
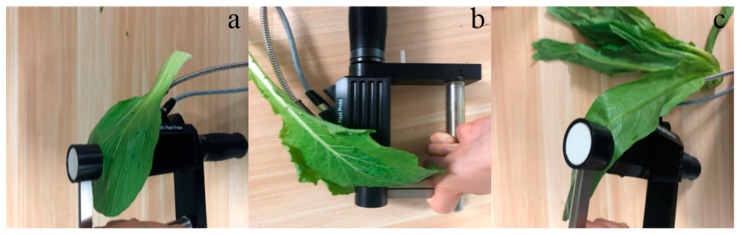
The measuring positions of the leaves for each plant species: (**a**) Pakchoi var. Shanghai Qing, (**b**) Chinese white cabbage, (**c**) Romaine lettuce.

For each leaf, two reflectance measurements were made on the adaxial and abaxial leaf surfaces using an ASD FieldSpec® HandHeld 2 spectrometer (Analytical Spectral Devices, Boulder, CO, USA). The spectral range of this spectrometer is 325–1075 nm, with a sampling interval of 1.4 nm and a spectral resolution of 3 nm. Due to the noise at the edge wavelengths of the spectrometer, only the reflectance range of 400–1000 nm was used in this study. Radiance was measured with an ASD leaf clip attached by a fore-optic probe for shielding the leaf from ambient light. The probe had a field-of-view of approximately 1 cm in diameter. The sample was clamped by the clips and irradiated by the beam from an internal incandescent light source with illumination perpendicular to the leaf. Each sample was scanned three times and averaged as the representative data for the sample. The measuring positions of leaves are shown in Figure 1. Similar measurements were made for a nearly 100% diffuse reflector (Spectralon, Labsphere, North Sutton, NH, USA) as a reference before every sample was measured. Spectral reflectance was computed by dividing the radiance reflected by the diffuse reflector.

### 2.3. Leaf Chlorophyll Extraction

To ensure the consistency between the reflectance and chlorophyll content of each leaf sample, three 0.6 mm diameter discs were cut from the approximate position on the leaf sample at which the reflectance measurement was taken. The discs were placed into a mortar with 0.6 g silica sand and 0.25 g calcium carbonate and then ground in the dark until the green color disappeared. Subsequently, the pigment mixture was transferred to a 50 mL volumetric flask with 96% ethanol to extract the chlorophyll, and then one part of the homogenous solution was removed for centrifuging in a plastic tube with a rotational speed of 927 g for 10 min. The supernatant of the pigment solution was separated from the plastic tubes and put into a cuvette for quantifying of chlorophyll content with a Lambda 900 UV/VIS spectrophotometer (PerkinElmer Inc., Waltham, MA, USA). Finally, the chlorophyll content was used in the empirical Equations (1)–(4) provided by Wintermans and De Mots (1965) [38].

(1) $$c_{a}(\mu g/mL)=13.7\times A_{665}m\mu -5.76\times A_{649}m\mu$$

(2) $$c_{b}(\mu g/mL)=25.80\times A_{649}m\mu -7.66\times A_{665}m\mu$$

(3) $$c_{total}(g/L)=c_{a}+c_{b}=6.10\times A_{665}m\mu +20.04\times A_{649}m\mu$$

(4) $$Chl(mg/m^{-2})=\frac{c_{total}(\mu g/mL)\times V(mL)}{S(cm^{2})}\times 10$$

where the *A*_665_ and *A*_649_ are the absorbance at the wavelengths of 665 and 649 nm, V is the volume of each sample solution and *S* is the area of each sample. The entire process of reflectance measurement and leaf chlorophyll extraction was conducted in a darkroom to avoid chlorophyll decomposition and keep the consistency between the reflectance and chlorophyll content measuring of each leaf sample.

### 2.4. Data Analysis

A reference leaf chlorophyll value was available for each reflectance measurement. The errors of leaf chlorophyll estimation were calculated for the dataset of pakchoi var. Shanghai Qing, Chinese white cabbage, Romaine lettuce as well as for three plant species combined.

One non-parametric regression method, PLS [39,40], and VIs were applied for leaf chlorophyll estimation. These methods are commonly used in remote sensing [3,24,25,41]. The PLS regression model is a method that specifies a linear relationship between a set of independent and response variables. In this study, PLS regression was used to model the correlation between leaf reflectance spectra (predictor variables) and leaf chlorophyll content (response variable). To ensure a reliable comparison between the two methods, the same calibration and validation data sets were used in both methods.

However, much of the information content within reflectance spectra maybe redundant and can be explained with fewer than 601 spectral bands [25]. Thus, before PLS modeling, an uninformative variable elimination PLS (UVE-PLS) approach was used for selection of the informative bands [24,25,26]. This method has been used previously to find informative spectral bands for LAI, leaf chlorophyll, and carotenoid content estimation [24,25]. UVE-PLS assists in reducing the data dimension by eliminating spectral data which are uninformative or redundant. When the UVE-PLS method is employed, a procedure of leave-one-out cross-validation was used to calculate the regression coefficients of all wavelengths (400–1000 nm). The non-informative bands were eliminated by the UVE-PLS method, which is based on the reliability parameter *c_wl_* and is computed using the PLS regression coefficients of each band as Equation (5):

(5) $$c_{wl}=\frac{\bar{b_{wl}}}{std\left(b_{wl}\right)}$$

where $\bar{b_{wl}}$ and $std\left(b_{wl}\right)\;$ are the average and the standard deviation of the PLS regression coefficients of all wavelengths (400–1000 nm), respectively. A low absolute value of the reliability parameter *c_wl_* means a low informative content band, and then these uninformative bands are eliminated from the PLS regression model [24,26]. The final PLS regression model for estimating chlorophyll utilized the retained bands. The PLS and UVE-PLS modeling was performed using the MATLAB R2014a software (The MathWorks, Inc., Natick, MA, USA).

The 30 VIs (Table 2), which were reported effective for leaf chlorophyll estimation at the leaf level were used. Some of the VIs were defined with specific formula and wavelengths as shown in Table 2, provided by the previous literature. One special VI named Modified Datt index (MDATT) [30], which was reported to be effective in estimating leaf chlorophyll content on both adaxial and abaxial surfaces, is an index with a specific formula, i.e., the ratio of the reflectance difference between different wavelengths as Equation (6), but without specific wavelength information.

(6) $${\mathrm{MDATT}\;\mathrm{index}(R}_{\lambda 1},R_{\lambda 2},R_{\lambda 3})=(R_{\lambda 3}-R_{\lambda 1})/\left(R_{\lambda 3}-R_{\lambda 2}\right)$$

where R_λ__1_, R_λ__2_, and R_λ__3_, are reflectance at different spectral bands. In order to obtain the optimal combination of bands, an exhaustive iteration of all the possible band combination was applied. Since the total 601 single-band reflectance (400–1000 nm) was used in this study, and the MDATT is a three-band index, 601^3^ combinations were calculated for optimizing the MDATT. The MDATT were fitted to the corresponding leaf chlorophyll content values by using a linear function commonly used for reflectance model versus plant physiological parameters relationships. The algorithms of the optimizing band combinations for the spectral and the regression analyses were created using a custom computer program in IDL software (Environmental Systems Research Institute, Inc., Redlands, CA, USA). The MDATT with the highest correlation coefficient was selected as optimum to estimate chlorophyll content for a specific dataset. The best performing relationships were validated using the validation datasets. The coefficient of determination (R^2^) and the root mean square error (RMSE) were calculated to compare the prediction abilities of those and the PLS regression model. The high R^2^ and low RMSE represented the higher precision and accuracy of the model in predicting the leaf chlorophyll content.

## 3. Results and Discussion

### 3.1. Reflectance of Adaxial and Abaxial Leaf Surfaces

**Figure 2:**
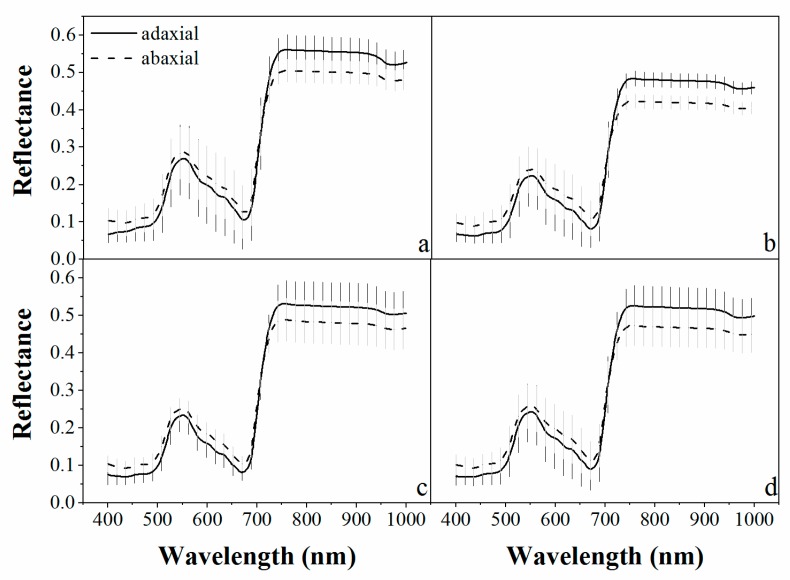
The average reflectance (full line for adaxial and dashed line for abaxial) and standard deviation (shadow region) of the adaxial and abaxial leaf surfaces for each plant species: (**a**) Pakchoi var. Shanghai Qing; (**b**) Chinese white cabbage; (**c**) Romaine lettuce, and (**d**) for the three species combined.

**Figure 3:**
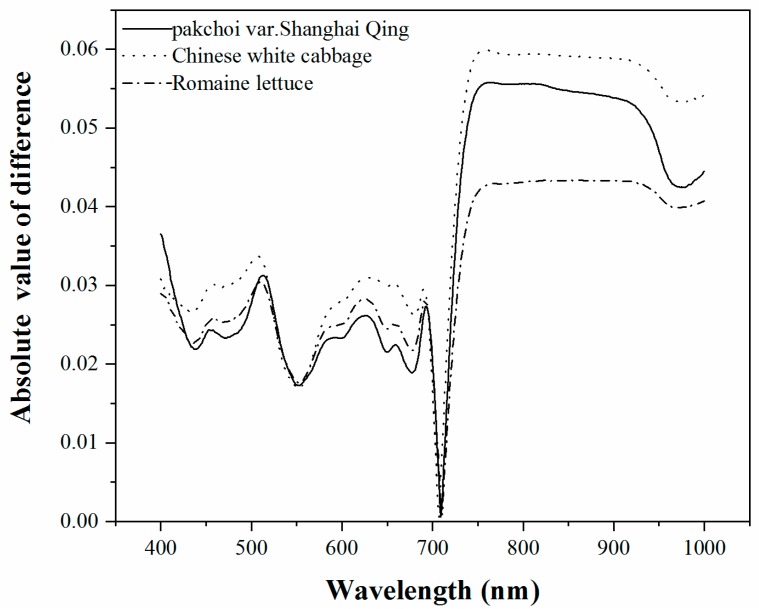
The absolute values of difference between the adaxial and abaxial leaf surfaces for each plant species.

The reflectance spectra of three different vegetable species measured from adaxial and abaxial leaf surfaces are presented in Figure 2. The trend of the spectral curves of the three vegetable species was similar. A significant difference was observed between the adaxial and abaxial reflectance for most wavelengths (P < 0.001). The reflectance of abaxial leaf surfaces was greater than that of the adaxial leaf surfaces in the visible spectral region (400–700 nm). The relative difference between the adaxial and abaxial reflectance was reversed from that of the spectral region of 700–750 nm (Figure 3). This observation was consistent with the results of Stuckens et al. [27] and Lu et al. [30,31]. It can be explained by the cross-section structures of bifacial leaves. The mesophyll cells at the adaxial leaf side are of palisade character, whereas those at the abaxial leaf side are of spongy structure [42]. The spongy cells with loose structure have more intercellular spaces that allow more scatter from the abaxial leaf side, while the compact structure of the palisade cells cannot scatter as much in the visible wavelengths. This may result in a higher reflectance from the abaxial leaf side and a lower reflectance from the adaxial side. In the NIR spectral region, the abaxial transmittance is often higher than that of the adaxial transmittance, which leads to a higher reflectance from the adaxial side and lower reflectance from the abaxial side [27].

The absolute values of the reflectance difference between the adaxial and abaxial leaf surfaces for the three vegetables species is shown in Figure 3. It was found that the reflectance difference in the visible region was less than in the NIR region. This is seemingly in conflict with the results of Lu et al. [30] which found that the reflectance difference in the visible region was larger than in the NIR region. In fact, the difference in NIR was almost equal for all of the plant species of both studies, but the reflectance in the visible wavelengths had the largest contrast. The adaxial reflectance of the woody species in Lu’s (white poplar and Chinese elm leaves) was much lower than that of the herbal species in this study. This may be caused by the different inner structures of the leaves in woody plants and annual herbaceous plants. Ivanova [43] had demonstrated that in the case of annual herbaceous plants, the ratios of spongy tissue prevailed over the palisade tissue. The higher percentage of spongy tissue that the vegetable species have may result in higher reflectance from the adaxial leaf side compared with those of the woody plants in the studies of Lu et al. and Stuckens et al. [27]. Much of the spongy tissue on the abaxial side of both the woody and vegetable species made little difference of the abaxial leaf reflectance between the woody plants and vegetables in this study.

The smallest reflectance difference between the adaxial and abaxial leaf surfaces for all of the vegetables was near 710 nm, which is located in the red-edge region. Thus, it is assumed that the spectral regions around 710 nm may be regarded as the least sensitive spectral band to leaf side structures. In addition, it is perhaps accurate to estimate chlorophyll content of leaves by the reflectance in the red-edge wavelengths when spectral information from the two leaf surfaces is considered. Of the three vegetables, the largest spectral difference between the two surfaces was observed in the Chinese white cabbage leaves, which may be due to the folds on the Chinese white cabbage.

### 3.2. Accuracy of Leaf Chlorophyll Estimation from the Reflectance Data from Two Leaf Surfaces

**Figure 4:**
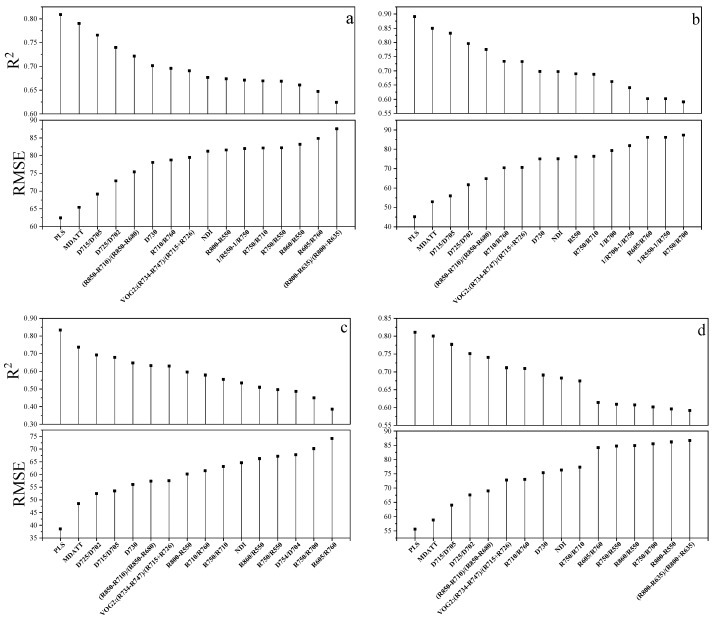
Comparison of the spectral indices and the PLS regression model for chlorophyll content estimation of (**a**) pakchoi var. Shanghai Qing, Modified Datt index (MDATT): (R_710_ − R_727_)/(R_710_ − R_734_); (**b**) Chinese white cabbage, MDATT: (R_703_ − R_732_)/(R_703_ − R_722_); (**c**) Romaine lettuce, MDATT: (R_712_ − R_744_)/(R_712_ − R_720_) and (**d**) the three species combined, MDATT: (R_705_ − R_732_)/(R_705_ − R_722_).

**Figure 5:**
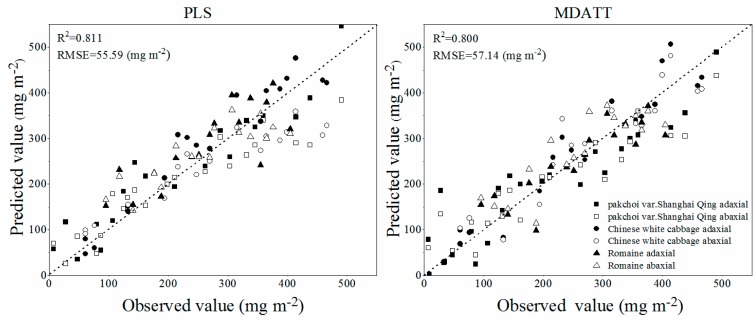
Predictive ability of the new MDATT index (R_705_ − R_732_)/(R_705_ − R_722_) and the PLS model.

The R^2^ and RMSE of the best 16 validation results of all of the tested methods, including 15 VIs and PLS regression model, are shown in Figure 4. The specific prediction results of PLS regression model and all VIs which were tested in this study are listed in Table 3 and Table 4. The PLS method had the highest accuracy of chlorophyll content estimation in terms of R^2^ and RMSE for each green leaves vegetable and three species combined. The PLS regression model, using the small number of spectral bands selected by UVE-PLS demonstrated the highest accuracy followed by the MDATT index in estimating the leaf chlorophyll content of pakchoi var. Shanghai Qing, Chinese white cabbage, and Romaine lettuce separately and also of the three vegetables combined. The PLS provided an RMSE of 62.44 mg m^−2^ (R^2^ = 0.809), 45.18 mg m^−2^ (R^2^ = 0.891), and 38.58 mg m^−2^ (R^2^ = 0.834) for pakchoi var. Shanghai Qing, Chinese white cabbage, and Romaine lettuce, respectively. The PLS also performed best in the dataset of three species combined, in which the RMSE was 55.59 mg m^−2^ (R^2^ = 0.811). The MDATT index gave the second best performance in both the individual plant species datasets and the three species combined. The RMSE was 65.41 mg m^−2^ (R^2^ = 0.790), 52.89 mg m^−2^ (R^2^ = 0.850), and 48.54 mg m^−2^ (R^2^ = 0.736) for pakchoi var. Shanghai Qing ((R_710_ − R_727_)/(R_710_ − R_734_)), Chinese white cabbage ((R_703_ − R_732_)/(R_703_ − R_722_)), and Romaine lettuce ((R_712_ − R_744_)/(R_712_ − R_720_)), respectively, and 57.14 mg m^−2^ (R^2^ = 0.800) for the three species combined ((R_705_ − R_732_)/(R_705_ − R_722_)). The results related to the MDATT index were consistent with those of Lu [30] in that it performed better than nearly all of the VIs tested. It has been shown in previous studies that MDATT was an effective index that is insensitive to the structures of adaxial and abaxial leaf surfaces for woody plant leaves. The results of this study presented that MDATT, formatted as the ratio of difference of reflectance is also available for leaf chlorophyll content estimation on herbaceous plant leaves, such as vegetables. However, it was a little inferior to the PLS method. The accuracy of green leaves vegetable chlorophyll estimation was slightly lower than previous studies, because in this study the validation results were provided instead of calibration results. Figure 5 shows the predictive ability of the MDATT index (R_705_ − R_732_)/(R_705_ − R_722_) and the PLS method for estimating the chlorophyll contents of the three vegetables combined. It can be found that the scatter points derived from PLS is closer to 1:1 line than those from MDATT. It is worth noting that the points with the leaves chlorophyll content smaller than 400 mg m^−2^ are more concentrated in the PLS model than in the MDATT index model. It was also discussed in Sims and Gamon [12] that MDATT index produced unstable results for leaves with very low chlorophyll content. In addition, the accuracy of chlorophyll estimation of Romaine lettuce using MDATT index was much lower than the other vegetables. This may be caused by the narrower chlorophyll content range of Romaine lettuce. Thus, MDATT index is considered more suitable for high chlorophyll content estimation and needs a wide range (low to high) of chlorophyll content to modeling. The PLS is a potentially more robust method to determine the leaf chlorophyll content of vegetables with the reflectance data from adaxial and abaxial surfaces and not affected by the chlorophyll content range in spite of having a little larger residual (>400 mg m^−2^).

Overall, although the best performing spectral index with the highest R^2^ was MDATT, which was in agreement with studies on the wooden or liana species [30,32], the PLS model was the more useful method in evaluating the leaf chlorophyll content when the reflectance of both leaf surfaces was considered, because with PLS, the regression model took more of the leaf chlorophyll sensitive spectral dataset into account than the spectral indices in a weighted viewpoint.

### 3.3. Spectral Band Selection for Estimating Leaf Chlorophyll Content with Reflectance from Two Leaf Surfaces

The two methods, PLS regression model and MDATT index, both demonstrated high accuracy in estimating the leaf chlorophyll content of three vegetable species separately as well as that of the three vegetables combined. Identifying the reliable spectral bands with different methods provides insight into the spectral features of reflectance specific to each species as well as those common to the three species, which have very different leaf structure. Identifying these spectral bands also allows the development of algorithms for estimating leaf chlorophyll with reflectance from two surfaces in these vegetables with no re-parameterization.

**Figure 6:**
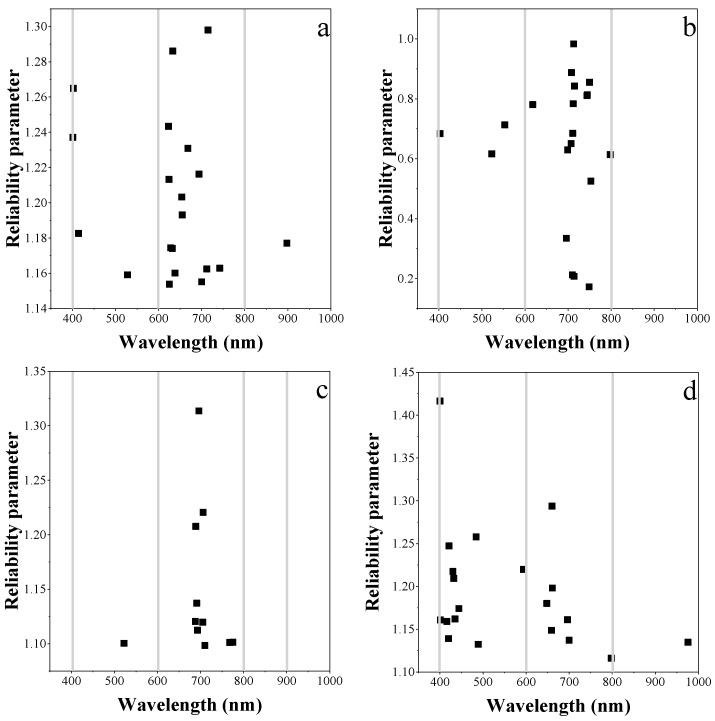
The band positions retained for estimating leaf chlorophyll by PLS regression model in (**a**) pakchoi var. Shanghai Qing; (**b**) Chinese white cabbage; (**c**) Romaine lettuce and (**d**) the three species combined.

UVE-PLS was applied to quantitatively evaluate the information content of reflectance data for leaf chlorophyll content estimation. The optimal bands selected for the individual vegetable species and the combination of the three are shown in Figure 6. For each single plant species, the red-edge and NIR wavelengths were the main informative bands. For the combination dataset, although the reliable bands were more dispersed than those of the individual species, the red-edge region was still retained. The informative spectral bands were able to achieve an RMSE below 55.59 mg m^−2^.

**Figure 7:**
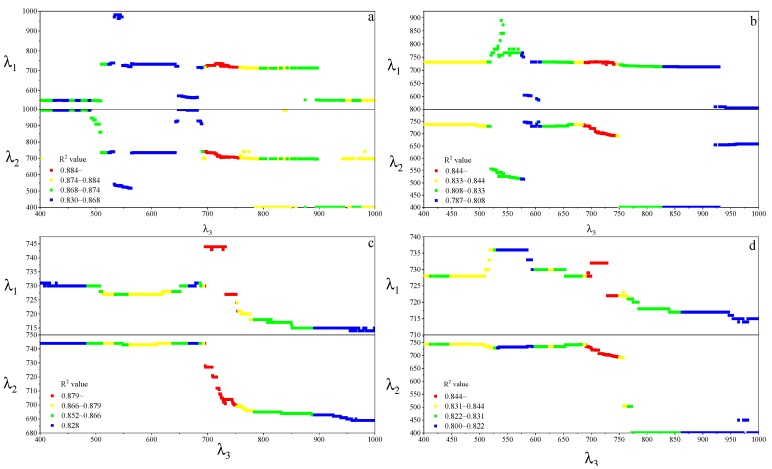
The band combination of λ_1_ and λ_2_ with the highest R^2^ when λ_3_ was fixed at every wavelength from 400–1000 nm. (**a**) Pakchoi var. Shanghai Qing; (**b**) Chinese white cabbage; (**c**) romaine lettuce, and (**d**) the three species combined.

**Figure 8:**
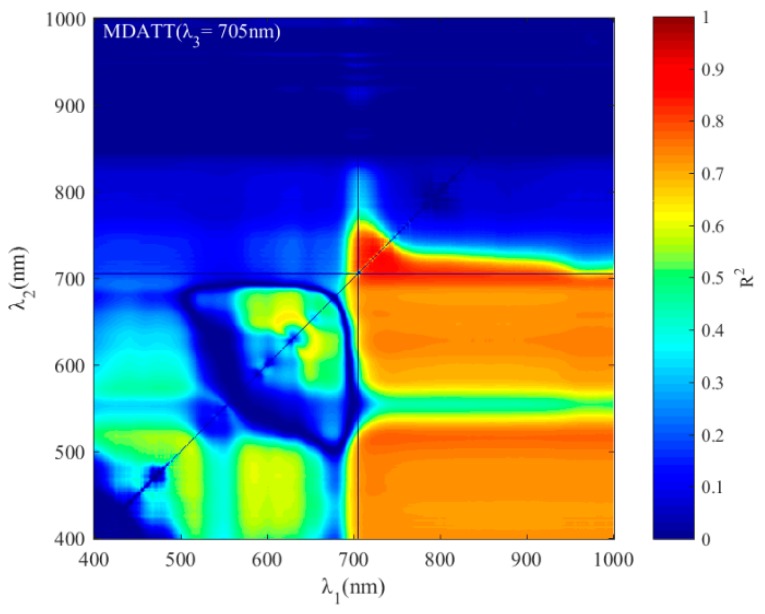
The dynamic variation of R^2^ when the λ_3_ was fixed at 705 nm for the dataset of three species combined.

For the MDATT index, the red-edge bands were also essential for leaf chlorophyll estimation in individual vegetable species and the three vegetables combined (Figure 7). The figures showed the band combinations with the highest coefficient of determination when λ_3_ was fixed on each band from 400–1000 nm. It was found that the MDATT index which had relatively high correlation (the 60 highest in red color) with the chlorophyll content owned very similar band combinations in any dataset studied in this research. The three bands used in the MDATT occurred on the red-edge region. For example, in the pakchoi var. Shanghai Qing dataset, the 718–736 nm for λ_1_, 703–742 nm λ_2_, and 697–757 nm for λ_3_ were the best band combinations; in the Chinese white cabbage dataset, the 722–733 nm for λ_1_, 692–738 nm λ_2_, and 685–745 nm for λ_3_ were the best band combinations; in the Romaine lettuce dataset, the 721–744 nm for λ_1_, 700–744 nm λ_2_, and 694–750 nm for λ_3_ were the best band combinations; and in the three vegetables combined dataset, the 722–732 nm for λ1, 694–742 nm λ_2_, and 690–750 nm for λ_3_ were the best band combinations. Figure 8 showed the dynamic variation of R^2^ when the λ_3_ was fixed at 705 nm for the dataset of three species combined. The combinations of 705, 722, and 732 nm for the MDATT index brought the RMSE below 57.14 mg m^−2^ for the three vegetables combined.

The consistency of the spectral bands retained by PLS and VIs is quite remarkable, indicating the robustness of the band selections. A very important result is that both methods tested were not species-specific for three different vegetables with different leaf structures. Thus, it is likely that the spectral bands selected in this study may be applicable in other vegetables to evaluate the leaf chlorophyll content.

It is instructive that the spectral band selection (red-edge regions) for MDATT consistently partly coincided with the bands selected by the PLS with informative spectral regions identified by UVE-PLS with a few exceptions. For the individual vegetation species, the red-edge bands were also selected by the UVE-PLS. However, the UVE-PLS selected bands for the combination of the three vegetable samples were a little far away from the optimal bands derived from the MDATT. It may be due to the fact that the red-edge reflectance could remove some effects of the different structures of leaf blades, but to remove the impacts of the difference between the vegetable species, it was necessary to combine more bands in the PLS analysis. Regardless, the consistency of band selection in MDATT and UVE-PLS showed that the red-edge region was effective in estimating the chlorophyll content of vegetables with reflectance from two leaf surfaces. Furthermore, the PLS method, including much more spectral bands, only improved the R^2^ and decreased the RMSE a little, which demonstrated that the bands outside the red-edge may only contribute to the improvement of the prediction of leaf chlorophyll content to a small degree.

## 4. Conclusions

The reflectance from the adaxial and abaxial surfaces of three leafy green vegetables was measured in this study. Two methods, namely, VIs and the PLS regression model, were used to estimate chlorophyll content. Although the bands were optimized using band optimum algorithms for some spectral indices, they did not outperform the PLS model for the derivation of the leaf chlorophyll content from both adaxial and abaxial reflectance. This is most likely due to the fact that the PLS model used more spectral bands than the spectral indices. PLS is a potentially useful method to evaluate the leaf chlorophyll content compared with the method of spectral indices when reflectance from both leaf surfaces is considered. In addition, the spectral regions selected by both the vegetation index of MDATT and PLS corresponded to the features of chlorophyll absorption, reflectance, and leaf scattering. The consistency of the spectral bands retained by MDATT and PLS indicated the robustness of the band selection. This finding would help estimate chlorophyll content in vegetable leaves accurately without re-parameterization of the algorithms. It is also an important step in the development of robust algorithms for remote sensing of vegetable biophysical parameters. 

## Figures and Tables

**Table 1:** Chlorophyll content (mg m^−2^) of three species vegetables extracted in this study.

Samples for Calibration	Total Chlorophyll Content (mg m^−2^)			Samples for Validation	Total Chlorophyll Content (mg m^−2^)		
	Minimum	Median	Maximum		Minimum	Median	Maximum
pakchoi var.Shanghai Qing (n = 45)	7.20	302.73	557.57	pakchoi var.Shanghai Qing (n = 21)	7.20	198.15	490.66
Chinese white cabbage (n = 43)	7.20	236.39	499.34	Chinese white cabbage (n = 17)	60.64	269.79	465.92
Romaine lettuce (n = 45)	76.06	276.70	446.18	Romaine lettuce (n = 17)	95.07	269.31	404.78

**Table 2:** Previously published spectral indices used in this study.

Spectral Index	References	Spectral Index	References
(R_850_ − R_710_)/(R_850_− R_680_)	Datt, 1999b	(R_800_ − R_650_)/(R_800_ + R_650_)	Blackburn, 1998b
D_754_/D_704_	Takebe and Yoneyama, 1989	PSNDb: (R_800_ − R_635_)/(R_800_ + R_635_)	Blackburn, 1998a
NDI: (R_750_ − R_705_)/(R_750_ + R_705_)	Gitelson and Merzlyak, 1994	VOG_2_: (R_734_ − R_747_)/(R_715_ + R_726_)	Vogelmann et al., 1993
D_730_	Richardson et al., 2002	1/R_700_−1/R_750_	Gitelson et al., 2003
R_672_/(R_550_*R_708_)	Datt, 1998	R_750_/R_700_	Lichtenthaler et al., 1996
R_860_/(R_550_*R_708_)	Datt, 1998	R_750_/R_550_	Lichtenthaler et al., 1996
1/R_700_	Gitelson and Merzlyak, 1996	1/R_550_−1/R_750_	Gitelson et al., 2003
R_800_/R_675_	Blackburn, 1998b	R_750_/R_710_	Zarco-Tejada et al., 2001
R_800_/R_650_	Blackburn, 1998b	R_710_/R_760_	Carter, 1994
PSSRb: B_800_/B_635_	Blackburn, 1998a	R_695_/R_420_	Carter, 1994
PSSRa: R_800_/R_680_	Blackburn, 1998a	R_605_/R_760_	Carter, 1994
R_672_/R_550_	Datt, 1998	R_550_	Carter, 1994
R_860_/R_550_	Datt, 1998	D_715_/D_705_	Vogelman et al., 1993
(R_800_ − R_675_)/(R_800_ + R_675_)	Blackburn, 1998b	D_725_/D_702_	Kochubey and Kazantsev, 2007
R_680_	Blackburn, 1998b	R_800_−R_550_	Buschman and Nagel, 1993

**Table 3:** Calibration and validation statistics of the partial least squares (PLS) regression models on the entire measurement spectra (400–1000 nm) for determination of the leaf chlorophyll content in each vegetable.

Vegetables	Calibration Dataset				Validation Dataset		
	N	PCs	R^2^	RMSE(mg m^−2^)	N	R^2^	RMSE(mg m^−2^)
pakchoi Var. Shanghai Qing	90	7	0.880	52.28	42	0.809	62.44
Chinese White Cabbage	86	16	0.894	42.20	34	0.891	45.18
Romaine Lettuce	90	8	0.879	38.66	34	0.834	38.58
All Combination	266	16	0.846	51.77	110	0.811	55.59

PCs: Number of latent variables; N: Number of samples.

**Table 4:** Validation statistics of spectral indices on the entire measuring spectra (400−1000 nm) for determination of the leaf chlorophyll content of each vegetable.

Spectral Index	Validation for Pakchoi Var. Shanghai Qing		Spectral Index	Validation for Chinese White Cabbage		Spectral Index	Validation for Romaine Lettuce		Spectral Index	Validation for Vegetables Combined	
	R^2^	RMSE (mg m^−2^)		R^2^	RMSE (mg m^−2^)		R^2^	RMSE (mg m^−2^)		R^2^	RMSE (mg m^−2^)
MDATT(R_710_ − R_727_)/(R_710_ − R_734_)	0.790	65.41	MDATT(R_703_ − R_732_)/(R_703_ − R_722_)	0.850	52.89	MDATT(R_712_ − R_744_)/(R_712_ − R_720_)	0.736	48.54	MDATT(R_705_ − R_732_)/(R_705_ − R_722_)	0.800	58.81
D_715_/D_705_	0.766	69.11	D_715_/D_705_	0.832	55.91	D_725_/D_702_	0.692	52.47	D_715_/D_705_	0.777	64.01
D_725_/D_702_	0.740	72.86	D_725_/D_702_	0.796	61.64	D_715_/D_705_	0.679	53.54	D_725_/D_702_	0.751	67.57
(R_850_ − R_710_)/(R_850_ − R_680_)	0.722	75.37	(R_850_ − R_710_)/(R_850_ − R_680_)	0.775	64.75	D_730_	0.648	56.11	(R_850_−R_710_)/(R_850_−R_680_)	0.741	68.99
D_730_	0.701	78.05	R_710_/R_760_	0.734	70.43	(R_850_ − R_710_)/(R_850_ − R_680_)	0.632	57.39	VOG_2_:(R_734_ − R_747_)/(R_715_ + R_726_)	0.711	72.78
R_710_/R_760_	0.696	78.77	VOG_2_: (R_734_ − R_747_)/(R_715_ + R_726_)	0.733	70.59	VOG_2_: (R_734_ − R_747_)/(R_715_ + R_726_)	0.629	57.56	R_710_/R_760_	o.710	73.00
VOG_2_: (R_734_ − R_747_)/(R_715_ + R_726_)	0.691	79.45	D_730_	0.698	75.04	R_800_ − R_550_	0.595	60.18	D_730_	0.691	75.34
NDI	0.677	81.21	NDI	0.698	75.06	R_710_/R_760_	0.578	61.42	NDI	0.682	76.34
R_800_ − R_550_	0.674	81.58	R_550_	0.689	76.07	R_750_/R_710_	0.554	63.13	R_750_/R_710_	0.674	77.31
1/R_550_ − 1/R_750_	0.671	81.95	R_750_/R_710_	0.688	76.30	NDI	0.533	64.59	R_605_/R_760_	0.614	84.16
R_750_/R_710_	0.669	82.14	1/R_700_	0.663	79.30	R_860_/R_550_	0.509	66.27	R_750_/R_550_	0.609	84.74
R_750_/R_550_	0.669	82.22	1/R_700_ − 1/R_750_	0.641	81.80	R_750_/R_550_	0.496	67.16	R_860_/R_550_	0.607	84.92
R_860_/R_550_	0.661	83.19	R_605_/R_760_	0.602	86.08	D_754_/D_704_	0.487	67.76	R_750_/R_700_	0.601	85.54
R_605_/R_760_	0.647	84.82	1/R_550_ − 1/R_750_	0.602	86.08	R_750_/R_700_	0.449	70.17	R_800_−R_550_	0.596	86.14
(R_800_ − R_635_)/(R_800_ + R_635_)	0.625	87.55	R_750_/R_700_	0.591	87.28	R_605_/R_760_	0.385	74.16	(R_800_ − R_635_)/(R_800_ + R_635_)	0.591	86.62
R_750_/R_700_	0.605	89.73	R_860_/(R_550_*R_708_)	0.589	87.50	1/R_550_ − 1/R_750_	0.374	74.80	R_550_	0.589	86.82
1/R_700_ − 1/R_750_	0.602	90.13	R_800_ − R_550_	0.571	89.42	R_860_/(R_550_ * R_708_)	0.357	75.83	1/R_700_ − 1/R_750_	0.587	87.02
PSSRb: R_800_/R_635_	0.599	90.40	(R_800_ − R_635_)/(R_800_ + R_635_)	0.570	89.55	1/R_700_ − 1/R_750_	0.333	7725	1/R_550_ − 1/R_750_	0.587	87.06
R_550_	0.596	90.80	R_750_/R_550_	0.564	90.14	(R_800_ − R_635_)/(R_800_ + R_635_)	0.329	77.46	(R_800_−R_650_)/(R_800_ + R_650_)	0.552	90.66
(R_800_ − R_650_)/(R_800_ + R_650_)	0.592	91.24	R_860_/R_550_	0.559	90.69	PSSRb: B_800_/B_635_	0.291	79.63	R_860_/(R_550_*R_708_)	0.548	91.05
R_860_/(R_550_*R_708_)	0.582	92.39	(R_800_ − R_650_)/(R_800_ + R_650_)	0.531	93.44	(R_800_ − R_650_)/(R_800_ + R_650_)	0.230	82.98	1/R_700_	0.537	92.21
R_800_/R_650_	0.579	92.69	PSSRb: B_800_/B_635_	0.447	101.56	R_550_	0.212	83.91	PSSRb: B_800_/B_635_	0.512	94.65
1/R_700_	0.544	96.49	R_695_/R_420_	0.446	101.62	R_800_/R_650_	0.209	84.07	R_800_/R_650_	0.472	98.43
(R_800_ − R_675_)/(R_800_ + R_675_)	0.458	105.14	R_680_	0.419	104.06	PSSRa: R_800_/R_680_	0.202	84.47	(R_800_ − R_675_)/(R_800_ + R_675_)	0.433	102.04
R_800_/R_675_	0.436	107.30	R_800_/R_650_	0.397	106.03	(R_800_ − R_675_)/(R_800_ + R_675_)	0.192	85.02	PSSRa: R_800_/R_680_	0.403	104.73
PSSRa: R_800_/R_680_	0.424	108.44	(R_800_ − R_675_)/(R_800_ + R_675_)	0.388	106.81	R_800_/R_675_	0.181	85.59	R_800_/R_675_	0.402	104.75
R_680_	0.397	110.92	D_754_/D_704_	0.344	110.54	1/R_700_	0.180	85.65	R_680_	0.396	105.30
D_754_/D_704_	0.361	114.21	PSSRa: R_800_/R_680_	0.333	111.48	R_672_/(R_550_*R_708_)	0.113	89.08	D_754_/D_704_	0.361	108.33
R_672_/R_550_	0.178	129.50	R_800_/R_675_	0.323	112.31	R_680_	0.055	91.92	R_695_/R_420_	0.190	121.92
R_695_/R_420_	0.082	136.89	R_672_/(R_550_*R_708_)	0.300	114.18	R_695_/R_420_	0.043	92.52	R_672_/(R_550_*R_708_)	0.159	124.24
R_672_/(R_550_*R_708_)	0.047	139.41	R_672_/R_550_	0.024	134.84	R_672_/R_550_	0.023	93.46	R_672_/R_550_	0.086	129.53
